# Awakened by a sleeping pill

**DOI:** 10.7554/eLife.01658

**Published:** 2013-11-19

**Authors:** Oluwaseun Akeju, Emery N Brown

**Affiliations:** 1 **Oluwaseun Akeju** is at the Department of Anesthesia, Critical Care and Pain Medicine, Massachusetts General Hospital, Boston, United States; 2 **Emery N Brown** is at the Department of Anesthesia, Critical Care and Pain Medicine, Massachusetts General Hospital, Boston, United States, and the Institute for Medical Engineering and Science, Department of Brain and Cognitive Sciences, Massachusetts Institute of Technology, Cambridge, United Statesenb@neurostat.mit.edu

**Keywords:** Consciousness, central thalamus, striatum, GABA-A, arousal, anesthesia, Human

## Abstract

Characteristic changes in brain activity accompany the paradoxical increase in alertness observed in some patients with severe brain injury when they are treated with the sleeping pill zolpidem.

**Related research article** Williams ST, Conte MM, Goldfine AM, Noirhomme Q, Gosseries O, Thonnard M, Beattie B, Hersh J, Katz DI, Victor JD, Laureys S, Schiff ND. 2013. Common resting brain dynamics indicate a possible mechanism underlying zolpidem response in severe brain-injury. *eLife* **2**:e01157. doi: 10.7554/eLife.01157

**Image** Brain activity in a patient with severe brain injury before (blue) and after (red) zolpidem

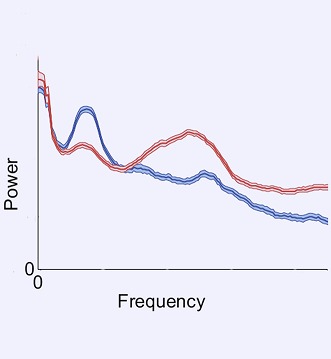

Treating patients with severe brain injuries remains one of the most challenging problems in neurology (Laureys and Schiff, 2012). However, six years ago, a serendipitous observation led to a possible new therapy (Brefel-Courbon et al., 2007). The idea for the therapy arose from the case of a 48 year-old woman who had been in a minimally conscious state for two years as a result of brain damage sustained in a suicide attempt. She could not move or feed herself, and could not speak, although she could understand speech. However, when she was given the sleeping medication zolpidem to treat insomnia, her family noticed that twenty minutes later she could communicate, eat, and move unassisted. By swapping her medications one by one, doctors confirmed that this strong arousal effect, which lasted for 2 to 3 hours, was due to zolpidem. Now, in *eLife*, Nicholas Schiff at Weill Cornell Medical College and co-workers—including Shawniqua Williams as first author—explore the neural basis of this effect and describe patterns of brain activity that could potentially be used to predict which patients will respond to this treatment (Williams et al., 2013).

It seems paradoxical that a sleeping medication can significantly improve cognitive and motor function in a minimally conscious patient. However, a similar phenomenon, known as paradoxical excitation, occurs in anaesthesiology whereby low doses of anaesthetics induce excitation rather than sedation (Brown et al., 2010). Zolpidem, in common with many of these anaesthetics, works by enhancing the activity of the brain’s inhibitory chemical transmitter, GABA. In a commentary accompanying the initial case report in 2007, Schiff and Jerry Posner offered a compelling hypothesis to explain the effect of zolpidem on the minimally conscious patient (Schiff and Posner, 2007). This was based partly on results from imaging studies, which showed that brain regions including the frontal cortex and the thalamus were highly active when the patient was on zolpidem and highly inactive when she was not (Brefel-Courbon et al., 2007).

Extensive damage to the cortex can lead to the loss of pathways between cortical regions, and between cortical and subcortical areas (Schiff and Posner, 2007). One such pathway consists of excitatory projections from the cortex to a subcortical structure called the striatum, which in turn sends inhibitory projections to a region called the globus pallidus. When not inhibited by the striatum, the globus pallidus inhibits the thalamus. The net effect is that loss of excitatory projections from the cortex after a severe brain injury can indirectly result in inhibition of the thalamus. Removing this inhibition is critical for restoring normal brain function because the thalamus is a major source of arousal inputs to the cortex. Zolpidem is known to be selective for a particular subtype of GABA receptors (GABA(A) alpha 1) which are expressed on inhibitory neurons in the globus pallidus. Schiff and Posner therefore proposed that zolpidem blocks the inhibitory inputs from this structure to the thalamus, thus allowing the thalamus to excite the cortex and help restore cognitive and motor functions.

Although functional improvement following zolpidem is well documented, it is also rare. Now, Williams et al.—who are based at various institutions in Belgium and the US—offer significant insights into the zolpidem paradox based on a study of three severe brain injury patients with strong arousal responses to the drug. The three patients, who had each experienced brain injury via a different mechanism, were tested on and off zolpidem using electrodes attached to the scalp to measure changes in the brain’s electrical activity. Off zolpidem, all three showed strong brain waves with an unusually low frequency (between 6 and 10 Hertz), which were most prominent over fronto-central regions of the scalp, and which were highly coherent within and between hemispheres. Zolpidem sharply reduced the strength and coherence of the 6-10 Hz activity, and led to an increase in the average frequencies of brain waves (15-30 Hz). These changes correlated with the improvements in alertness seen in the patients.

By linking their clinical observations to in vitro and in vivo neurophysiology studies, Williams et al. reasoned that the 6-10 Hz oscillations probably arise from the intrinsic membrane properties of the damaged neurons in the cortex. They suggest that the brain waves become coherent because those brain areas with residual electrical activity that remain connected will have a tendency to begin firing together at a common frequency. They therefore interpret the coherent 6-10 Hz brain waves as a marker of reserve capacity that could be recruited to restore function, for example, through the use of drugs such as amantidine and zolpidem, or devices such as deep brain stimulation and transcranial magnetic stimulation. Zolpidem probably works by breaking up the coherence in the network through the mechanism outlined above. Williams et al. thus offer new insights into the therapeutic use of zolpidem and suggest a potential diagnostic and prognostic brain wave signature that is easy to measure.

Along with the scientific findings, other lessons can be learned from this study. The integration of clinical practice, pharmacology, formal behavioral testing and basic neurophysiological reasoning into an informative observational study establishes a paradigm which others in clinical neuroscience should emulate. These findings also support the need for a larger clinical trial to evaluate the effects of zolpidem on the recovery of brain function in patients with severe brain injury. Given that zolpidem is only effective in a small number of patients, and there is extensive variation in the expression of GABA receptor subtypes between individuals (Kang et al., 2011), these studies should include genetic profiling. Future work will help to identify those patients most likely to benefit from zolpidem and the best therapeutic regimen to use, as well as the diagnostic and prognostic value of the 6-10 Hz brain waves.

**Figure 1.**
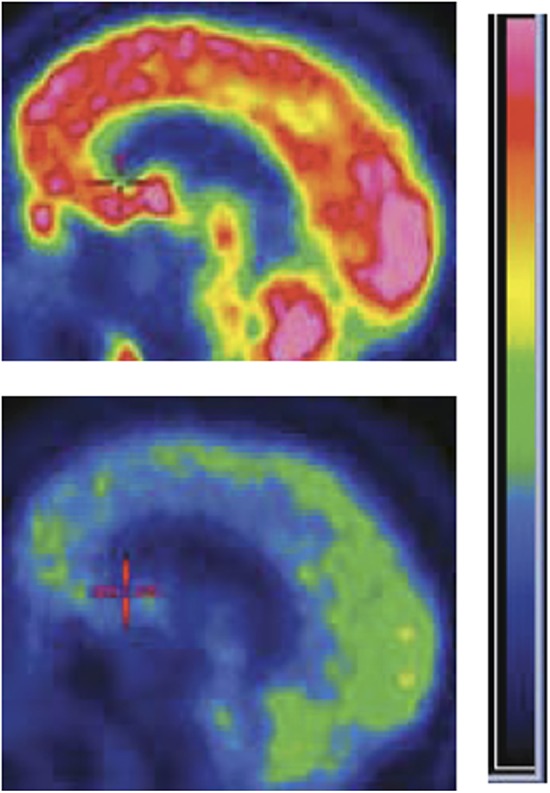
Zolpidem increases brain activity in some patients with severe brain injury. Positron emission tomography (PET) images of the brain of a patient with severe brain injury before (bottom) and after (top) taking the sleeping medication zolpidem. The brighter colours represent increased rates of glucose metabolism, which are used as a proxy of brain activity.

Figure credit: Image adapted from Figure 8 in Williams et al., 2013.
